# A Technique for Obtaining Balanced and Functional Occlusion Through Modified Wax-Path Record

**DOI:** 10.7759/cureus.44149

**Published:** 2023-08-26

**Authors:** Durga Bhavani Kanaparthi

**Affiliations:** 1 Department of Prosthodontics and Crown & Bridge, Drs Sudha and Nageswara Rao Siddhartha Institute of Dental Sciences, Vijayawada, IND

**Keywords:** occlusal adjustments, face bow transfer, wax-path record, alu wax, modelling plastic compound, eccentric movements, removable prosthodontics, single complete denture, balanced and functional occlusion, functionally generated path

## Abstract

A difficult treatment problem exists to construct a complete denture as opposed to an unmodified natural dentition. A harmonious occlusion is difficult to achieve when malposed, supra-erupted or missing teeth are present in the opposing arch. This incurs compromised stability and retention, failure of the prosthesis and patient discomfort. The main aim of this article is to construct a functionally stable removable complete denture against an unmodified dentition using a functionally generated pathway (FGP) technique. The FGP technique refers to the “three-dimensional static expression of dynamic tooth movement.” The functional movements made by the patient are utilized in this technique to have a harmonious occlusion with minimal intra-oral occlusal adjustments. The FGP technique can be used in the fabrication of a single crown, fixed partial dentures, removable partial dentures, complete dentures and dental implant prosthesis. This clinical report describes a method for developing the FGP occlusion for the patient having a completely edentulous maxillary arch against Kennedy’s class IV edentulous mandibular arch.

## Introduction

Fabrication of a single complete denture against an unmodified natural dentition or reconstructed dentition is a tough thing for the clinician to obtain balanced and functional occlusion due to the fixed position of natural dentition [1,2]. The functionally generated pathway (FGP) of occlusion relies on eccentric excursions, recorded with the help of a plastic medium [3-6]. Meyer termed this technique as the chew-in technique, and since then the procedure has been modified by various research [7-9].

The research of Pankey and Mann with improved materials has extended the prosthodontic applications of the functionally generated path technique [10]. The procedure is simplified by the usage of newer materials and techniques [10]. Stansbury introduced a technique of functional chew-in based on the work of Meyer [1,2]. Meyer developed this concept to overcome the undesirable factors acting on the prosthesis and to overcome the deficiencies of articulators [2,8]. Over the years, several names have been given to this technique such as “functional bite technique,” “functional chew-in technique,” “generated path technique” and “cuspal tracing technique” [10].

The functionally generated pathway is formed by engraving the functional wax medium by the opposing cusps with functional border movements of the mandible such as protrusive and lateral movements which are eccentric mandibular movements. The mandible translates in a forward and downward direction during protrusive movement. Lateral movement occurs when one condyle rotates within the temporomandibular fossa and the other condyle translates forward, inward and downward [11]. This FGP technique is contraindicated when the denture space is inadequate and when the desired jaw movements are difficult to achieve. All rehabilitation procedures in the opposing dental arch should be completed prior to the construction of the denture [12]. This article presents a case report of prosthodontic rehabilitation of a patient with a completely edentulous maxillary arch against unmodified natural teeth using the functionally generated pathway technique.

## Case presentation

A 58-year-old woman approaches the Department of Prosthodontics for the fabrication of dentures. History revealed that she is dissatisfied with the previous complete denture due to poor retention and stability. On clinical examination, the patient presents with an edentulous upper arch and a partially edentulous lower arch with Kennedy’s class IV classification.

A removable partial denture is fabricated in the mandibular arch prior to the construction of the maxillary single complete denture. Once all the desired procedures are established, preliminary impressions of the upper and lower arches are made. Primary impression of the mandibular arch is made with irreversible hydrocolloid material (Chromatex, DPI, India), and primary impression of the maxillary arch is made with a modeling plastic compound (Pyrax Co., Madhya Pradesh), and diagnostic casts are obtained. A special tray is constructed over the diagnostic cast of the maxillary arch using an auto-polymerizing acrylic resin material (DPI Cold cure; Dental Products of India, Mumbai) with the tray borders 2mm short of the sulcus. Border molding is performed using low-fusing green stick compound (DPI Pinnacle, Tracing sticks Dental Products of India), and a wash impression is made up of an irreversible rigid impression material (zinc oxide eugenol) (Pyrax Co., Madhya Pradesh).

**Figure 1:**
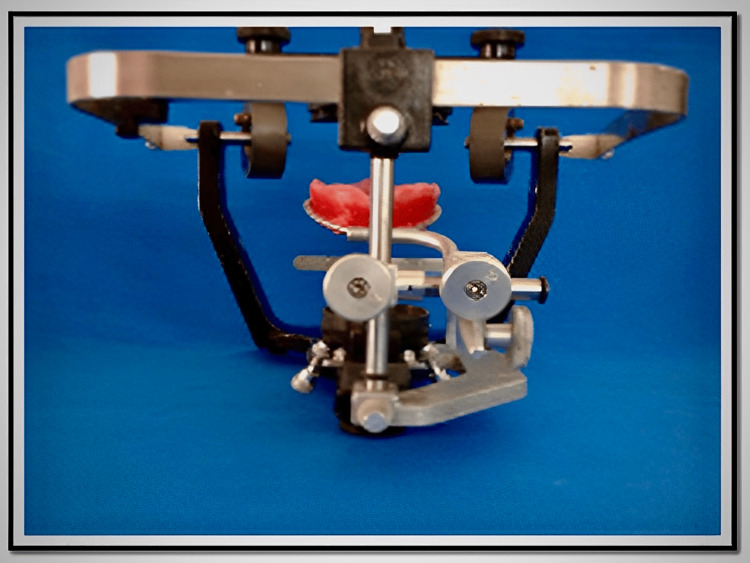
Face-bow record transferred to the articulator

Beading and boxing of the definitive impression is made and poured with a type III dental stone (Blue Kalstone, Madhya Pradesh). After setting, the master cast is separated from the impression. The post-dam area is obtained by scraping the cast. The casts are trimmed and indexed. Two denture bases are fabricated on the maxillary master cast using an auto-polymerizing resin material. Two occlusion rims, made up of modeling wax (Y-Dent, Madhya Pradesh) on one denture base and modeling plastic compound on another denture base, are fabricated. A lip support, lip fullness and desired vertical dimension of occlusion are to be established in the wax occlusion rim. A desired vertical dimension of occlusion is established where the phonetics, esthetics and inter-occlusal space are used as guides, and at this established vertical dimension, the centric relation is recorded. Then the face-bow transfer record is made clinically (Hanau^TM^ Springbow). Using the face-bow transfer record, the wax occlusion rim is transferred to the semi-adjustable articulator (Hanau^TM^ Wide-Vue) (Figure 1).

**Figure 2:**
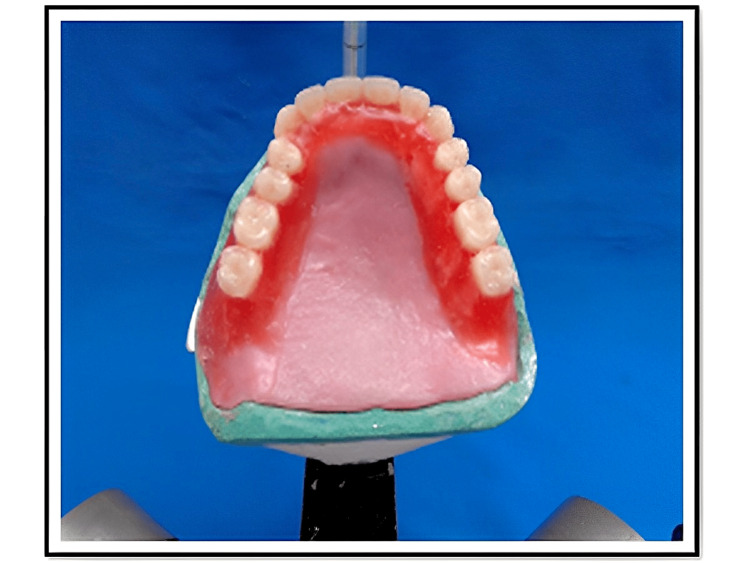
Trial denture The teeth arrangement is done based on the glass plate relation, and this trial denture is inserted into the patient's mouth to confirm phonetics, esthetics and preview the patient's smile.

Both the casts are mounted on a semi-adjustable articulator using a contingent jaw relation record to incur the functional movements of the mandible. A trial denture is fabricated and inserted during the try-in (Figure 2).

**Figure 3:**
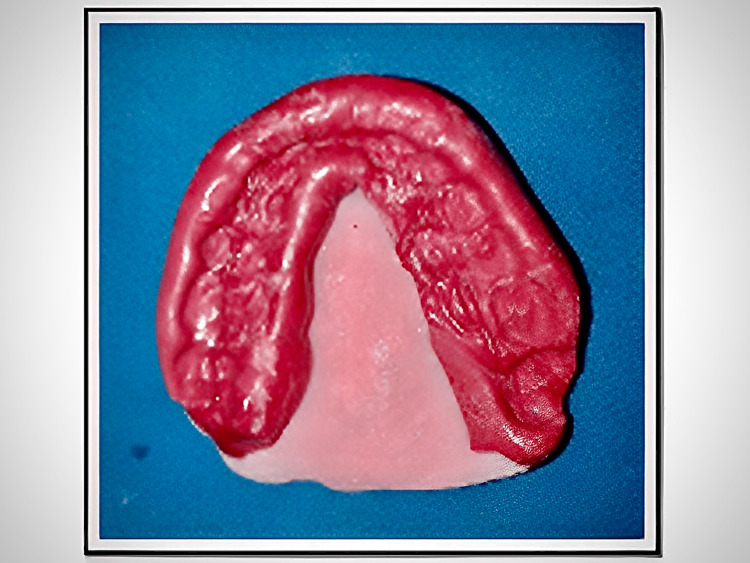
Modeling plastic occlusion rim with indentations

The modeling plastic occlusion rim is retained on the denture base by placing retentive pins below the material. The modeling plastic occlusion rim is warmed and placed on the master cast, and the articulator is closed to have indentations of the opposing arch. The plastic occlusion rim is trimmed to establish 2mm of space between the rim and the mandibular anterior teeth. Reference points are placed to maintain the desired vertical dimension of occlusion throughout the functionally generated path technique. The buccal and lingual cusp tips of the posterior teeth should not come in contact with the plastic occlusion rim in the articulator (Figure 3).

**Figure 4:**
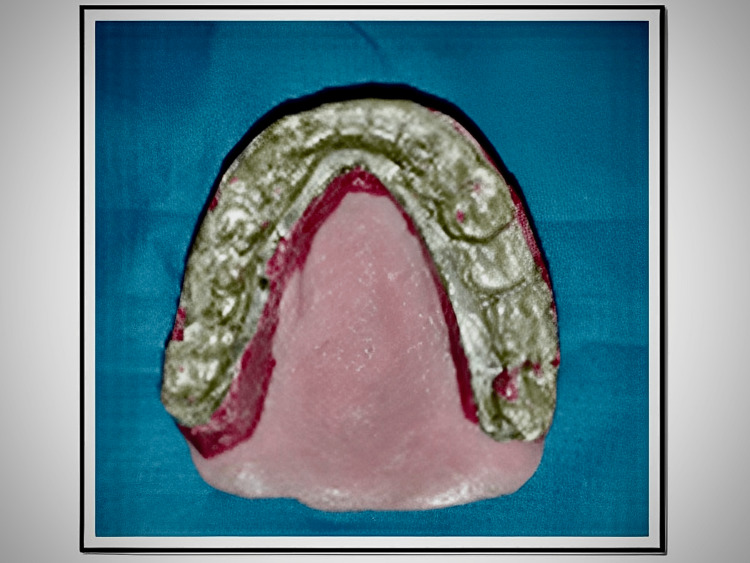
Functionally generated path It is the wax-path record, wherein we can observe the markings made by the mandibular posterior teeth when the patient executed protrusive and lateral excursions. The mandibular anterior teeth are used as a stop for the centric position.

The modeling plastic occlusion rim is inserted into the patient’s mouth, and a tentative jaw relation record is verified. Alu wax (Alu wax Dental Products Co., USA) is added to the full width and length of the occlusion rim for the functionally generated path procedure. Both the materials are tempered for 10 to 15 seconds for the flow of Alu wax into the indentations present in the modeling plastic occlusion rim. This whole is placed into the patient’s mouth and trained to close first into centric occlusion, and the mandibular anterior teeth tracings are recorded in registration wax. These tracings serve as stops in the future to record centric relations. The centric, protrusive and lateral excursion movements are recorded. Firstly, protrusive excursion movement is recorded by guiding the patient to bring the mandible into the forward position from the retruded position, approximately 6mm, anterior to the posterior position. Secondly, lateral excursion movements are recorded by guiding the patient to move the mandible to either right or left about 5mm and move back to the retruded position, and these movements produce a functionally generated path record (Figure 4).

**Figure 5:**
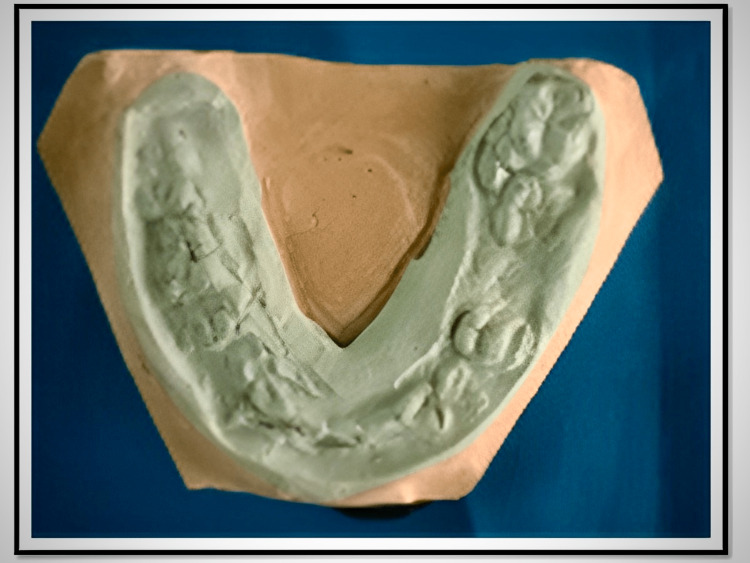
Stone core It is the positive replica of the patient's functionally generated wax-path record. By using this stone core, the occlusion in the trial denture is adjusted.

The generated wax-path is poured with type III dental stone into the wax pathways traveled by the cusps to a depth of 10-15 mm. The wax is removed from the stone core after the set of the stone (Figure 5).

**Figure 6:**
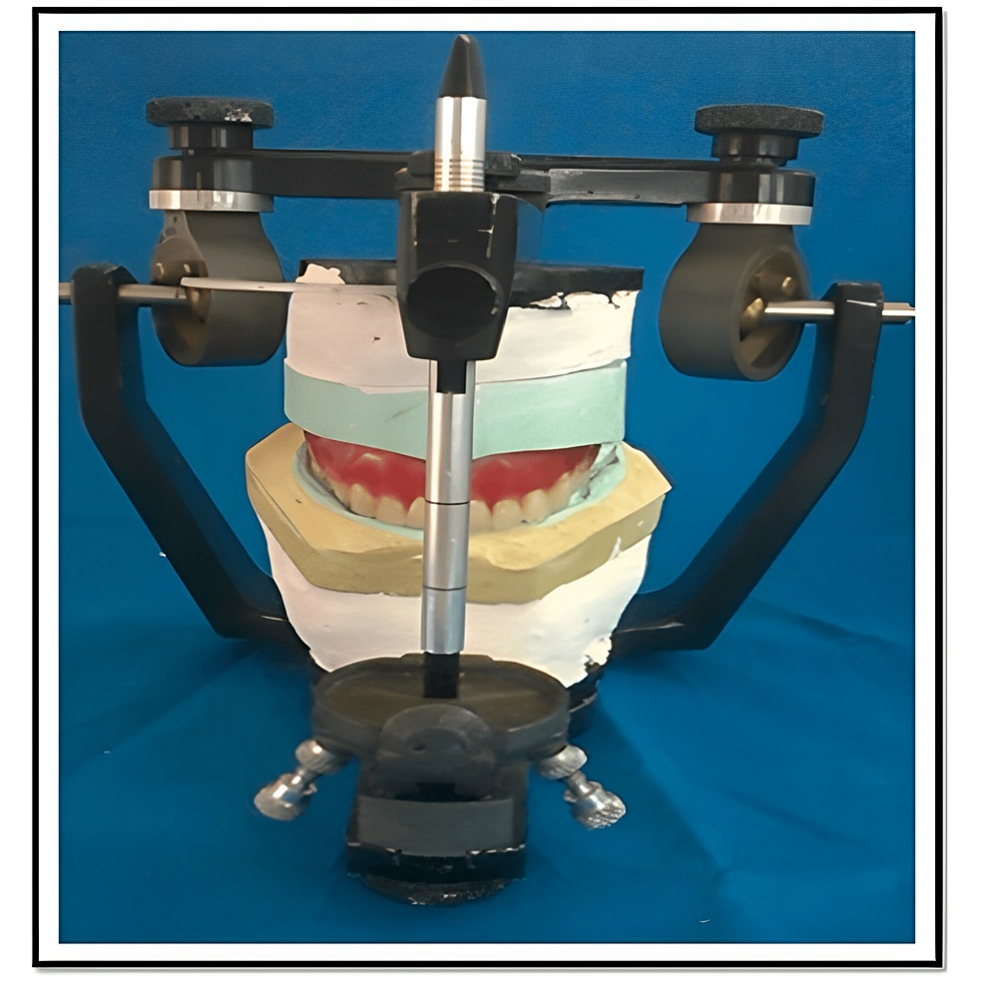
Stone core attached to the articulator

The stone core is placed in the articulator in the position of the mandibular cast at the desired vertical dimension, and the occlusal contacts of the trial denture with the stone core are evaluated and grinded to fit the generated path record on the stone core (Figure 6).

**Figure 7:**
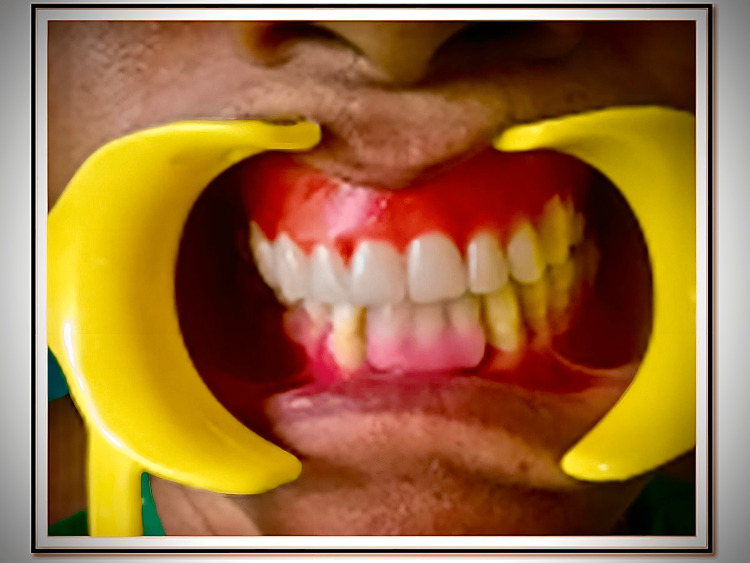
Try-in Try-in of the trial denture is done in the patient's mouth after removing all the occlusal discrepancies in the centric and eccentric positions. Hence, a balanced and functional occlusion is established in both centric and eccentric excursions.

Articulating papers are placed between the trial denture and the stone core after which the protrusive and lateral excursion movements are performed in the articulator. Hence, markings are made on the trial denture which are used as an indicator for premature occlusal contacts, protrusive and lateral interferences. All these interferences are grinded until the incisal guide pin touches the incisal guide table, establishing a maximum bilaterally balanced and functional occlusion in both centric and eccentric excursions (Figure 7).

The trial denture is placed in the articulator to check occlusion prior to flasking. The trial denture is flasked, dewaxed and processed in the normal conventional manner. After cooling, the denture is deflasked and remounted in the articulator to check for any processing errors. Any interferences in the occlusion and vertical dimension are put right by doing occlusal adjustments of teeth. Then proper finishing and polishing of the denture is done. Finally, the denture is inserted into the patient’s mouth without any deflective occlusal contacts with a balanced and functional occlusion that characterizes the functionally generated path occlusion. The post-insertion instructions are given to the patient.

## Discussion

A technique for the fabrication of a single complete denture as opposed to non-modified natural dentition or reconstructed dentition is illustrated. In this procedure, the FGP method is incurred for producing a harmonious occlusion. This technique is simple, reliable and highly versatile. The functionally generated path establishes harmonious occlusion without any deflective occlusal contacts. In this method, the functionally generated path is achieved using a semi-adjustable articulator because this articulator simulates and incorporates the patient’s protrusive and lateral excursion movements. The clinical and laboratory procedures are done in the normal conventional manner [2,13]. The grinding surfaces of the prosthetic teeth fit into the stone core that represents the generated path record. This stone core record looks somewhat bizarre as it represents the dynamics of mandibular movements.

In this technique, the patient’s border movements of the mandible (both centric and eccentric) are recorded. The functionally generated pathway procedure produces harmonious occlusion with minimal processing errors compared to usual denture fabrication techniques. The FGP technique is utilized in the fabrication of a single crown, fixed partial dentures, removable partial dentures, complete dentures and dental implant restorations.

The Pankey-Mann philosophy of occlusal rehabilitation gains balance in eccentric excursions [10]. Vig described a technique which is similar to Stansbury technique, but Vig used a flattened appendage of a resin material in the grinding surfaces of the teeth [14]. Sharry described a technique of using softened wax in the maxillary rim [15]. The functional paths of the cusps of the mandibular teeth are recorded by protrusive and lateral chewing movements [15]. In this clinical report, a balanced and functional occlusion is achieved in the trial denture itself, against the stone core before processing of denture. This gives more freedom of movement for the teeth to achieve balanced and functional occlusion. Only minor selective grinding of teeth is done to correct deflective occlusal contacts after denture processing.

Waxes must have a particularly hard consistency so that, if subject to excessive pressure, they will break rather than alter their shape. In previous articles, modeling wax was used for the same technique. Modeling wax is an extremely soft wax that cannot maintain its plastic state at room temperature. In some previous articles, auto-polymerizing resins were used as recording material. But these resins have the disadvantage that they inevitably shrink during polymerization making it difficult to reposition them on casts. So some special wases are to be used for craniomandibular recordings. 

In this case report, a special type of wax called Alu wax is used along with a modeling plastic compound. Alu wax contains metallic (aluminum) elements that increase the wax coefficient of temperature, prolonging the duration of its plasticity and allowing the clinician an extended working time. It provides the heat retention properties needed for efficient modeling. Alu wax is a reliable material that can be efficiently used as a recording material. Hence, the mandibular movements of the patient are recorded accurately using the modeling plastic compound and Alu wax recording medium.

Articulators are capable of reproducing, to a greater or lesser degree, mandibular movements. Articulators are useful in analyzing functional relationships between maxillary and mandibular arches. In previous articles, small non-adjustable articulators and mean value articulators are used. However, they have the disadvantage that non-adjustable articulators result in improper prosthetic rehabilitation, and mean value articulators are used only in simple prosthetic reconstructions. The mean values reproduced by the mean value articulator do not correspond to the average clinical values.

In this case report, the Hanau Wide-Vue articulator is used which is a semi-adjustable articulator. This articulator is capable of recording and reproducing the angle of eminence, the Bennett angle, the anterior guidance, the intercondylar distance and the patient's mandibular centric and eccentric movements. Hence, the materials and instruments used in this technique add information to the existing literature.

## Conclusions

The functionally generated pathway technique is indicated for a single crown, fixed partial dentures, removable partial dentures, complete dentures, single complete denture as opposed to natural or reconstructed teeth and dental implant restorations. This method utilizes the patient’s masticatory system to develop occlusion, and this technique is simple, accurate and reliable. The functionally generated pathway technique is practical, fundamental and functional. Harmonious occlusion (balanced and functional) which is achieved by this FGP technique is very important because if any occlusal discrepancies are present, it leads to poor stability, poor retention of the denture and patient discomfort. In this technique, a special type of wax called Alu wax and a semi-adjustable articulator are used which add information to the existing literature.

## References

[1] Stansbury CB (1951). Single denture construction against a non-modified natural dentition. J Prosthet Dent.

[2] Bruce RW (1971). Complete dentures opposing natural teeth. J Prosthet Dent.

[3] (2005). The glossary of prosthodontic terms. J Prosthet Dent.

[4] Dawson PE (1974). Long centric. Evaluation, Diagnosis and Treatment of Occlusal Problems, 2nd  Ed.

[5] Dawson PE (1974). Anterior guidance. Evaluation, Diagnosis and Treatment of Occlusal Problems, 2nd  Ed.

[6] Dawson PE (1974). Functionally generated path techniques for recording border movements intraorally. Evaluation, Diagnosis and Treatment of Occlusal Problems, 2nd  Ed.

[7] Meyer FS (1951). Dentures: causes of failures and remedies. J Prosthet Dent.

[8] Meyer FS (1959). The generated path technique in reconstruction dentistry. Part I. Complete dentures. J Prosthet Dent.

[9] Meyer FS (1959). The generated path technique in reconstruction dentistry. Part II. Fixed partial dentures. J Prosthet Dent.

[10] Pankey LD, Mann AW (1960). Oral rehabilitation. Part II. Reconstruction of the upper teeth using a functionally generated path technique. J Prosthet Dent.

[11] Manary DG, Holland GA (1984). Evaluation of mandibular movement recording and programming procedures for a molded condylar control articulator system. J Prosthet Dent.

[12] Rudd KD, Morrow RM (1973). Occlusion and the single denture. J Prosthet Dent.

[13] Rudd KD (1964). Processing complete dentures without tooth movement. Dent Clin North Am.

[14] Vig RG (1964). A modified chew-in and functional impression technique. J Prosthet Dent.

[15] Sharry JJ (1962). Complete Denture Prosthodontics, 3rd Ed.

